# Designing clinical AI for patient-centered support beyond the visit: the PACT framework for health systems

**DOI:** 10.1038/s44401-026-00116-w

**Published:** 2026-08-05

**Authors:** Saif Khairat, Charles Safran

**Affiliations:** 1 Carolina Health Informatics Program, University of North Carolina at Chapel Hill, Chapel Hill, NC USA; 2 Sheps Center for Health Services Research, University of North Carolina at Chapel Hill, Chapel Hill, NC USA; 3 Lineberger Comprehensive Care Center, University of North Carolina at Chapel Hill, Chapel Hill, NC USA; 4 Harvard Medical School, Boston, MA USA

**Keywords:** Business and industry, Computational biology and bioinformatics, Health care, Mathematics and computing, Scientific community

## Abstract

Clinical AI has advanced rapidly for bounded in-visit tasks such as prediction, documentation, and message generation, yet many costly failures in care occur outside the encounter when follow-up, handoffs, and communication break down. We argue that the next challenge for clinical AI is not only better task performance, but better operational follow-through across the care journey. We propose the PACT framework (Patient-centered, AI-enabled Continuity and Timely action) that reframes clinical AI as a health system function designed to support continuity, completion, escalation, and equity across pre-visit, visit, and post-visit care. The PACT framework specifies the operational elements required for accountable action, including ownership, communication channels, confirmation rules, escalation pathways, and outcome measures. We illustrate its practical use through post-visit coordination, a high-impact setting in which health systems can test workflow integration, monitoring, and tiered human support.

## Background

Clinical AI has advanced most rapidly in settings where data are structured, workflows are controlled, and clinicians are already present, such as ambient documentation and radiology reports generation. Yet many of the health system’s most consequential failures occur outside the encounter: during care transitions, between appointments, after hours, and in the home, when patients and families must interpret plans, coordinate next steps, monitor symptoms, and decide when to seek help. These times are when continuity succeeds or breaks down, and where health systems often rely on patients and care partners to absorb essential coordination work with little formal support.

For decades, pioneers such as Warner Slack argued for patient-centered computing and described patients and families as underused resources in healthcare^1,2^. In the era of clinical AI, that insight must be extended from participation to operational support. A patient may leave a visit with an accurate diagnosis and an evidence-based treatment plan, yet safe execution still depends on many actions beyond the clinic: obtaining medications, arranging follow-up, understanding warning signs, aligning transportation and caregiving, managing symptoms at home, and escalating concerns when conditions change. The challenge is operational. Care plans frequently fail not because decisions were absent, but because the health system lacks reliable mechanisms to convert decisions into completed, confirmed, and supported actions across people, settings, and time.

Care coordination failures cost the US health system an estimated $27 to $78 billion annually^3^. Communication breakdowns contribute to nearly half of malpractice claims^4^. Approximately 20% of patients do not fill a newly prescribed chronic disease medication within 30 days^5^. Advanced cancer patients who miss a first oncology appointment face substantially shorter survival and higher emergency utilization than those who attend^6^. Much of this burden arises not from isolated diagnostic or treatment errors, but from fragmentation across the care journey: missed follow-up, unclosed loops, unclear ownership, delayed escalation, and uneven support for patients and care partners after the visit. Innovation and investment have concentrated on episodic clinical tasks, while a large share of preventable harm, waste, and inequity accumulates in longitudinal coordination failures.

Clinicians also spend substantial time documenting and navigating electronic health records, often at the expense of direct patient engagement^7^. Tools that improve documentation efficiency may reduce clerical burden, but they do not resolve the larger system challenge of ensuring that clinical decisions reliably translate into completed actions after the encounter^8,9^. For clinical AI to deliver meaningful value at the health system level, its target must expand beyond optimizing discrete tasks within visits.

### Health systems blind spots

The historical focus of clinical AI on diagnosis, prediction, and other bounded tasks is understandable. From early expert systems to contemporary applications in radiology, pathology, risk stratification, documentation, and message generation, much of clinical AI has been designed around functions that fit existing workflows and can be evaluated using narrow performance metrics^10,11^. These advances can improve efficiency and technical performance, but they do not directly address the failures that most often undermine continuity of care.

The distinction between recognition failures and operational failures matters because many of the most consequential breakdowns in care are not failures of recognition. A patient may receive an accurate diagnosis and an appropriate plan during the visit, yet still experience harm when medications are not obtained, appointments are not completed, symptoms worsen without response, or care partners are left uncertain about what to do next. In such situations, the unit of failure is not the isolated clinical decision, but the chain of actions required to translate that decision into completed, coordinated, and supported care.

Patient-centered care provides a well-established standard for what better care should achieve, including respect for patient values, coordinated and integrated care, understandable information, emotional support, and inclusion of families and caregivers when appropriate^12^. Yet these principles remain difficult to operationalize in routine care because health systems often lack the infrastructure to assign responsibility, confirm completion, and respond when plans begin to break down^13^. Patients and care partners, therefore, absorb much of the coordination burden, not as a matter of design, but as a consequence of fragmented workflows, workforce strain, and weak accountability across transitions.

Current AI efforts rarely define that coordination problem explicitly. Many tools are intended to improve documentation efficiency, prediction, or communication, but they do not specify who owns the next step, how task completion is confirmed, when escalation should occur, or how failures are surfaced before patients fall through the cracks^14,15^.

The narrow, task-level framing also carries equity risks. AI-enabled coordination strategies that assume portal access, digital literacy, English proficiency, stable housing, transportation, or available caregiving support may work best for the patients who are already easiest to reach. Prior experience with biased clinical algorithms has shown that optimization without attention to structural conditions can reproduce or worsen disparities^15^.

Many current AI tools provide real value in bounded settings, particularly when reducing administrative burden or improving task performance.^16^. Task-level gains, however, are not sufficient if the broader follow-through architecture remains weak. Better notes, better predictions, and faster messages do not automatically produce continuity, comprehension, or accountable action after the visit. Without designs that explicitly support patients, care partners, and clinical teams across transitions, AI may optimize parts of care while leaving the highest burden of coordination work unresolved.

The concern that AI will de-personalize care is legitimate, yet it often conflates automation of coordination with automation of the human relationship. The PACT framework redirects AI to absorb that mechanical coordination work, confirming follow-through, tailoring communication, and escalating when plans break down, so that clinicians can return their attention to the person in front of them.

Ambient documentation tools, diagnostic imaging AI, risk stratification models, and patient-facing large language models each represent meaningful advances in bounded clinical tasks^8,9,14,17,18^. None reliably specifies who acts on the output, how completion is confirmed, or when escalation should occur. Performance is measured at the point of generation, the note, the prediction, the message, rather than at the point of consequence: whether the care plan was understood, executed, and followed through safely after the visit.

We therefore argue that the central blind spot in current clinical AI is not simply underperformance at the point of care, but underinvestment in the mechanisms that ensure follow-through beyond the clinical encounter. Closing that gap is the purpose of the PACT framework proposed here.

### The PACT framework for health systems

We propose the PACT framework, a patient-centered AI-enabled Continuity and Timely support framework for health systems, focused on how clinical AI can help support patients and care partners before, during, and after the visit through clear guidance, assigned actions, monitoring, confirmation, and timely escalation. The framework’s purpose is to define the minimum operational elements required for AI-supported care to produce accountable action: a clear objective, a designated user or users, an assigned owner, a communication channel, a confirmation step, an escalation trigger, and outcomes by which performance can be judged.

The central premise is that clinical AI should not end at output generation. A note, recommendation, risk score, or message draft has limited value in isolation; each requires a delivery mechanism that assigns responsibility, supports execution, detects breakdowns, and initiates follow-up when the plan is not carried through. The PACT framework, therefore, treats AI as part of a broader orchestration layer within care delivery, one that connects patients, care partners, clinicians, navigators, and digital infrastructure across time rather than serving only a single encounter.

**Fig. 1:**
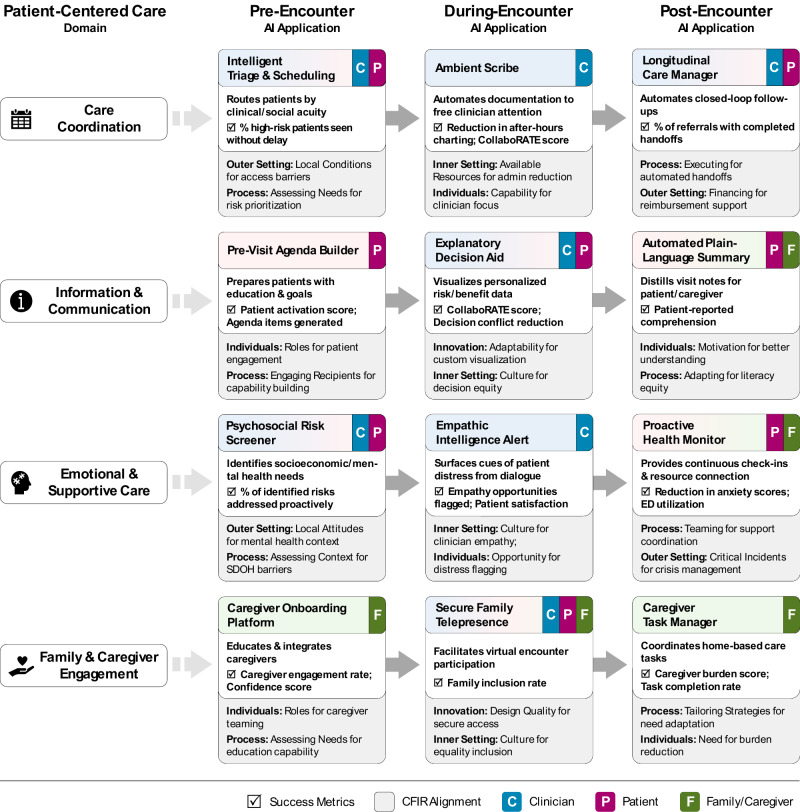
The PACT framework maps key care functions across the pre-visit, visit, and post-visit stages of care. For each domain, it specifies the operational elements required for accountable follow-through, including the coordination objective, primary users, responsible owner, communication channel, confirmation rule, escalation trigger, and outcomes for evaluation. The framework is intended to guide design, implementation, and assessment of clinical AI around continuity, completion, and equity rather than isolated task performance. Shaded elements indicate the post-visit exemplar discussed in the text.

Figure 1 organizes this orchestration logic across patient-centered care domains, including coordination, information and communication, emotional support, and family and caregiver engagement. For each cell, the framework specifies the coordination objective, the primary actors involved, the person or team responsible for task completion, the channel through which communication occurs, the rule by which completion is confirmed, and the condition under which escalation to additional support is required. In this way, the framework shifts the design question from “What can the model generate?” to “How will the health system ensure that the right action happens, is confirmed, and is supported if it does not?”

The PACT framework also embeds a multi-level evaluation model. Process measures include task assignment, completion, time to confirmation, failed outreach, and appropriate escalation when follow-through breaks down. Patient and care partner measures include understanding of the plan, confidence in next steps, coordination burden, and ability to carry out tasks safely at home. Clinical and system measures include missed follow-up, delayed treatment, emergency visits, avoidable readmissions, and other downstream consequences relevant to the use case. These measures should be stratified by language, digital access, health literacy, caregiving support, and related social risk factors so that health systems are judged not only by average performance, but by whether continuity improves for those most at risk of being missed.

The same orchestration logic applies across stages of care, even though the tasks differ. In the pre-visit stage, the framework supports structured intake that captures symptoms, goals, barriers, and caregiving constraints in a form that can shape visit preparation. During the visit, the framework supports shared understanding through agenda-setting, plain-language explanation, clarification of trade-offs, and documentation of who will do what next. In the post-visit stage, the framework becomes most operationally visible by translating the care plan into assigned tasks, reminders, confirmation prompts, and escalation pathways delivered through channels that match patient circumstances, including telephone and text when portal-based strategies are insufficient. Across all stages, coordination improves when each task has an owner, a time horizon, a confirmation rule, and a response plan for noncompletion.

Several design principles follow from this framework. First, different users need different outputs, so AI-generated content should be role-specific rather than generic: patients and care partners need understandable next-step guidance, clinicians need concise decisions and ownership summaries, and navigators or nurses need work queues that surface unresolved tasks and escalation needs. Second, patient-centered design should not be equated with self-service. Many patients and families can act with the right support, but some situations require a rapid transition from automated assistance to human intervention. The framework, therefore, assumes tiered support, in which AI can assist with coordination when appropriate but reliably hands off to a navigator, nurse, pharmacist, or clinician when risk is elevated, tasks remain incomplete, or confusion persists. Third, because orchestration succeeds or fails within real delivery systems, implementation must be designed from the outset for workflow fit, staffing capacity, reimbursement conditions, technical integration, and equitable access. Fourth, patients and families should be engaged as active participants in co-developing, implementing, and evaluating closed-loop systems, not only as recipients of coordination.

### A practical starting point: post-visit coordination

**Fig. 2:**
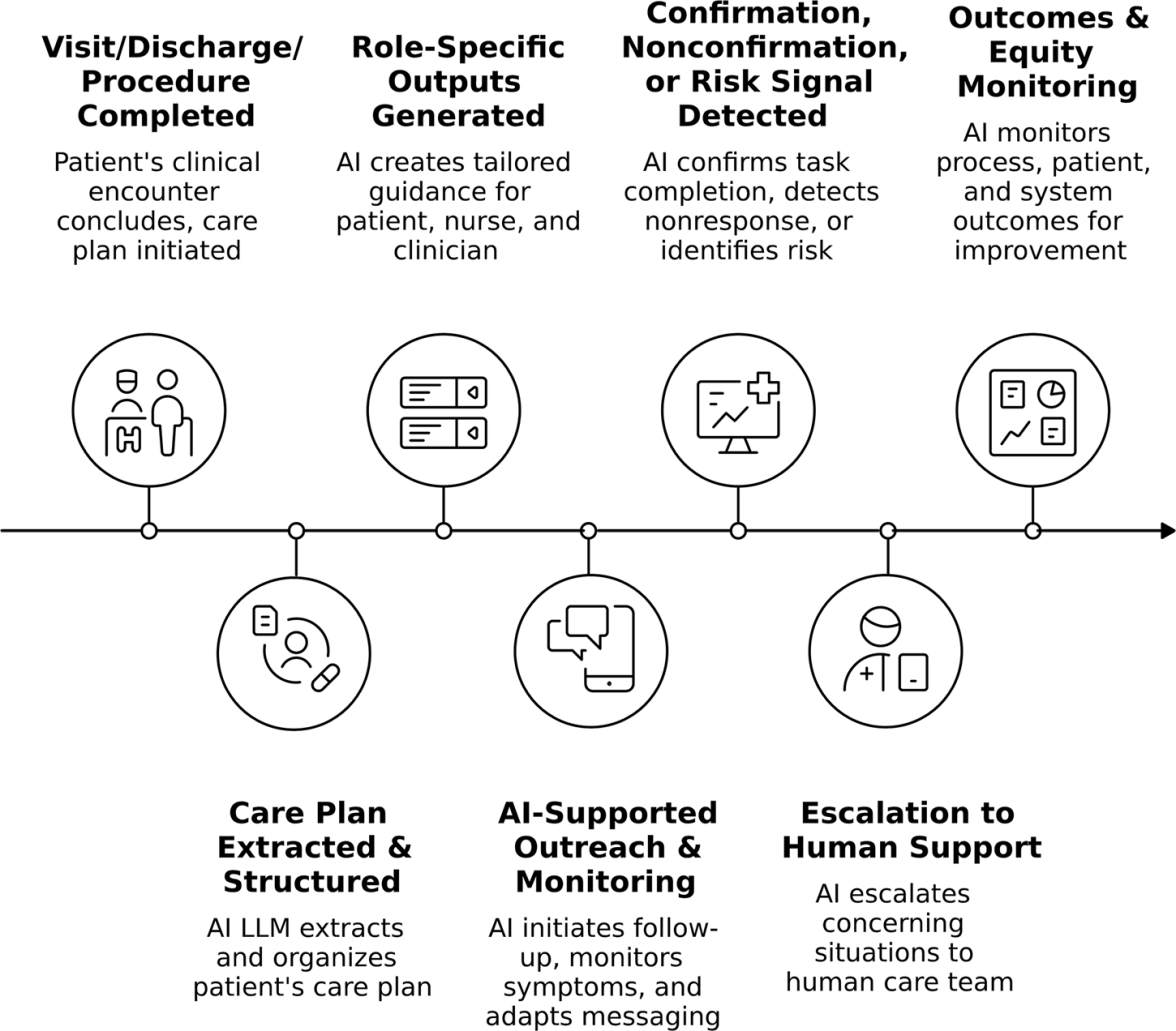
Post-visit PACT framework workflow for health systems. Following a visit, discharge, or procedure completion, the care plan is translated into actionable tasks and role-specific outputs for patients, care partners, and clinical teams. The AI-enabled coordination layer supports patient-facing guidance, remote monitoring, communication, confirmation of task completion, and escalation when follow-through fails or risk signals emerge. The workflow emphasizes owner assignment, time frame, confirmation, and escalation, while monitoring outcomes and equity to inform redesign and ongoing oversight.

A practical starting point for the PACT framework is the post-visit period after discharge, a procedure, or a high-risk ambulatory visit, where coordination failures are common, consequential, and measurable (Fig. 2). At this stage, patients and care partners must interpret instructions, start medications, arrange follow-up, monitor symptoms, and decide when to escalate concerns, often with limited support. “Clinically important and operationally visible” repeats “common, consequential, and measurable” from the preceding clause. Simplify to: “Post-visit coordination is a feasible, high-impact entry point for health systems implementing clinical AI.”

Consider a hypothetical patient, a 62-year-old man with newly diagnosed colorectal cancer living in a rural county 2 h from his oncology center. Completion of his first chemotherapy visit triggers the workflow. AI translates the care plan into role-specific outputs: plain-language recovery instructions for the patient, a task list for the caregiver, and an action-oriented work queue for the nurse navigator. An AI-enabled remote monitoring tool tracks symptom reporting between visits, while a patient-facing LLM delivers reminders and fields follow-up questions through text. Each task carries a named owner, a target time frame, a confirmation rule, and an escalation pathway.

When the monitoring tool detects no symptom log entry by hour 36, the system escalates automatically to the navigator, who conducts a telehealth check and identifies an unmanaged nausea episode preventing medication adherence. Early escalation resolves the gap before an avoidable emergency department visit occurs, illustrating how the PACT framework converts a care plan into a completed, confirmed, and supported action across distance and time.

Success in post-visit coordination should be measured not by message volume or chatbot use, but by closed-loop task completion, reduced patient confusion, fewer missed follow-up visits, safer home recovery, and lower downstream utilization.

Post-visit coordination offers a bounded setting in which health systems can test workflow integration, staffing, escalation thresholds, and monitoring before extending the same logic to pre-visit and in-visit care.

### Governance and safety in deployment

Because these systems shape what happens after the visit, governance must extend beyond predeployment validation to ongoing oversight in real-world use. Health systems should define who is responsible for monitoring task completion, missed confirmations, failed outreach, inappropriate escalation, and subgroup performance over time. In this context, safety events include not only incorrect information but also silent coordination failures such as incomplete medication starts, missed follow-up, unresolved symptom reports, and nonresponse from patients or care partners. The PACT Framework should therefore be deployed with clear thresholds for human review, routine audit of completion and escalation patterns, and explicit processes for redesign or rollback when performance declines, or disparities widen.

### Boundary conditions and next steps

The PACT framework is a conceptual guide rather than a validated standard, and several implementation challenges warrant acknowledgment. Workforce preparation is a primary concern: clinicians, navigators, and administrative staff would require structured training to adopt new role assignments and escalation protocols under conditions of existing strain. A phased rollout, beginning with post-visit coordination and integration into existing quality improvement infrastructure, could reduce that burden. Patient preparation carries equal weight, as closed-loop coordination depends on patients and care partners’ understanding what is expected of them and through which channel, requiring plain-language onboarding and proactive identification of those who need human-assisted rather than automated support from the outset.

Reimbursement poses a structural barrier, as current fee-for-service models do not consistently reimburse the navigation, monitoring, and follow-up work the framework requires. Value-based arrangements and remote patient monitoring codes offer partial coverage, but broader adoption will depend on payment reform that rewards follow-through rather than encounter volume. Equity risks demand deliberate design: workflows built around portal access, digital literacy, or English proficiency will reach the most connected patients first. The framework’s tiered support model is intended to mitigate that risk, but health systems must actively monitor subgroup performance and redesign workflows when disparities widen.

Empirical evidence supports the framework’s constituent mechanisms. Oncology navigation programs combining digital monitoring with structured nurse escalation have reduced acute care utilization in randomized settings^19^. Telehealth-based post-discharge coordination has produced 30-day readmission rates significantly below matched benchmarks^20^. AI-enabled virtual navigation has nearly doubled cancer screening completion among previously nonadherent patients^21^. No published study has yet evaluated the full orchestration model as proposed here. Future work should test the framework prospectively across diverse care settings to determine where closed-loop coordination improves continuity, reduces coordination burden, and advances equity in practice.

## Conclusion

Closed-loop care orchestration, operationalized in the PACT framework, reframes clinical AI as a health system function rather than a stand-alone tool. The approach aims to ensure that care plans are translated into completed, confirmed, and equitably supported actions through clear ownership, timely escalation, and ongoing monitoring of follow-through. By judging success in terms of continuity, accountability, and completion rather than isolated task performance, health systems can deploy clinical AI to better support patients, care partners, and care teams throughout the care journey.

## Data Availability

No datasets were generated or analysed during the current study.

## References

[1] Sands, D. & deBronkart, D. Warner Slack: “Patients are the most underused resource”. *BMJ* **362**, k3194 (2018).

[2] Crotty, B. H. et al. Information sharing preferences of older patients and their families. *JAMA Intern. Med.* **175**, 1492–1497 (2015). 26147401 10.1001/jamainternmed.2015.2903

[3] Shrank, W. H., Rogstad, T. L. & Parekh, N. Waste in the US health care system: estimated costs and potential for savings. *JAMA* **322**, 1501–1509 (2019). 31589283 10.1001/jama.2019.13978

[4] Humphrey, K. E. et al. Frequency and nature of communication and handoff failures in medical malpractice claims. *J. Patient Saf.* **18**, 130–137 (2022). 35188927 10.1097/PTS.0000000000000937

[5] Franklin, J. M. et al. Time to filling of new prescriptions for chronic disease medications among a cohort of elderly patients in the USA. *J. Gen. Intern. Med.* **33**, 1877–1884 (2018). 30054889 10.1007/s11606-018-4592-6 PMC6206334

[6] Delgado Guay, M. O. et al. Characteristics and outcomes of advanced cancer patients who miss outpatient supportive care consult appointments. *Support Care Cancer* **22**, 2869–2874 (2014). 24771301 10.1007/s00520-014-2254-8

[7] Khairat, S. et al. Association of electronic health record use with physician fatigue and efficiency. *JAMA Netw. Open* **3**, e207385 (2020). 32515799 10.1001/jamanetworkopen.2020.7385 PMC7284310

[8] Dai, T., Kvedar, J. C. & Polsky, D. Policy brief: ambient AI scribes and the coding arms race. *npj Digit. Med.* **8**, 780 (2025). 41444833 10.1038/s41746-025-02272-z PMC12738533

[9] Van Linschoten, R. C. A., van Loon, C. M., Joanknecht, L. & Bischoff, E. W. M. A. Ambient scribe in general practice: a multi-perspective before-after longitudinal mixed-methods study. *npj Digit. Med.* **9**, 299 (2026). 41772212 10.1038/s41746-026-02454-3 PMC13065737

[10] Shortliffe, E. H. & Buchanan, B. G. A model of inexact reasoning in medicine. *Math. Biosci.* **23**, 351–379 (1975).

[11] Slack, W. V. et al. A computer-based physical examination system. *JAMA* **200**, 224–228 (1967). 6071437

[12] World Health Organization (WHO). Integrated people-centred care. [12/3/2025]. https://www.who.int/health-topics/integrated-people-centered-care#tab=tab_1 (2016).

[13] World Health Organization. Framework on integrated, people-centred health services. [January 27, 2026]. https://apps.who.int/gb/ebwha/pdf_files/wha69/a69_39-en.pdf (2016).

[14] Steel, P. A. D. et al. Learning health system strategies in the AI era. *npj Health Syst.* **2**, 21 (2025). 42527455 10.1038/s44401-025-00029-0 PMC13354208

[15] Wiens, J. et al. Do no harm: a roadmap for responsible machine learning for health care. *Nat. Med.* **25**(9), 1337–1340 (2019). 31427808 10.1038/s41591-019-0548-6

[16] Lukac, P. J. et al. A randomized-clinical trial of two ambient artificial intelligence scribes: measuring documentation efficiency and physician burnout. Preprint at *medRxiv*10.1101/2025.07.10.25331333 (2025).

[17] Gu, Z. et al. Radiology workflow assistance with artificial intelligence: establishing the link to outcomes. *J. Am. Coll. Radiol.* **23**, 389–398 (2026). 41106573 10.1016/j.jacr.2025.10.018

[18] Zaretsky, J. et al. Generative artificial intelligence to transform inpatient discharge summaries to patient-friendly language and format. *JAMA Netw. Open* **7**, e240357 (2024). 38466307 10.1001/jamanetworkopen.2024.0357 PMC10928500

[19] Gervès-Pinquié, C. et al. Impacts of a navigation program based on health information technology for patients receiving oral anticancer therapy: the CAPRI randomized controlled trial. *BMC Health Serv. Res.* **17**, 133 (2017). 28193214 10.1186/s12913-017-2066-x PMC5307879

[20] Horman, S. F. et al. Virtual transition of care clinics and associated readmission rates: 3-year retrospective cohort study. *JMIR Med. Inform.* **13**, e73495 (2025). 40987450 10.2196/73495 PMC12504893

[21] Moadel, A. B. et al. AI virtual patient navigation to promote re-engagement of U.S. inner city patients nonadherent with colonoscopy appointments: a quality improvement initiative. *J. Clin. Oncol.* **42**, 100–100 (2024).

